# Diet and Service in Mental Hospitals

**Published:** 1912-09-14

**Authors:** 


### DIET AND SERVICE IN MENTAL HOSPITALS.

### Dr. J. Francis Dixon on an Experiment at West Humberstone.

In a recent issue we alluded briefly to certain improvements in the quality and variety of diet which the Committee of the Leicester Borough Mental Hospital, West Humberstone, had decided to introduce. Knowing the value to other mental hospitals of such experiments, our Commissioner visited West Humberstone to learn from Dr. Dixon the nature and object of the innovation. During the course of the interview he said :—

"An important preliminary step was taken by the Committee shortly before my appointment last year, in the form of a modification of the weekly routine of diets. Under the old system the same meal occurred on the same day every week, so that a patient who had been in residence for a short time would know, for instance, as every Friday came round exactly what to expect for dinner. As a result of a tour of inspection of other mental hospitals, the Committee, under the chairmanship of Alderman Dr. George Clifton, J.P., had determined on further improvements in the quality and variety of the food, and I myself had already devoted some attention to the subject. Thanks to the able co-operation of the Clerk and Steward, Mr. Sydney C. Leak, the difficulties involved in ministering to individual tastes are being largely overcome. These changes are already meeting with much appreciation from the more sensible patients, and the increase in their happiness and contentment has brought about a marked improvement in their general health."

"What conclusions had you previously arrived at?"

"In a short paper, dealing with the "Use of Sedatives in Insanity," read before the Medico-Psychological Association and published in the *Journal of Mental Science* about three years ago, brief reference was made to the distinction between *stimulating* and *sedative* diets, and it was remarked that it might be well if the diet tables of our institutions for the treatment of mental diseases were revised in accordance wih the progress made in the science of dietetics. The specific suggestion for the use of a number of set diets, which was referred to in The Hospital, had its basis in this contention."

"The question had also its administrative side?"

"Undoubtedly; here, as in other institutions where a stock diet on a weekly basis is the rule, one saw a large amount of avoidable waste. There being little scope for individual taste and need, patients are often found rejecting their meals altogether or in part, and the result is that a large quantity of food finds its way into the swill-tub. There is also another source of waste. The provision of extras becomes necessary, and it is difficult to keep a grip on extras. A variety of set diets practically covering all cases does away with the necessity for extras."

"You say the set diets are now developing?"

"Yes, but they have only been getting into shape during the last few months. There are two ordinary diets—meat and vegetarian—in the latter are included milk, eggs, and cheese, besides the strictly vegetable foods. These are changed weekly, and frequently include one diet which has not been used before. As well as these diets there are four special set diets, which are still in the experimental stage. Each diet is tested by its popularity with the patients. We are able in this way to approach the type of diet to which the patients are accustomed in their own homes. In the case of epileptics, who form an example of those requiring non-stimulating food, a vegetarian diet is now the rule. A good type of the vegetarian dinner is that which consists of vegetable broth and a really well-cooked and appetising cabinet pudding with white sauce. A return of the waste from each ward is carefully made."

"Is beer used as part of a diet?"

"No, it has long been discontinued. Some breweries exist in mental hospitals, and in many have only recently been given up. The psychological aspect of diet is not entirely forgotten. On Sundays dinner consists of roast beef or roast pork for all able to eat it, thus deferring to a well-established British custom. The increase in the consumption of vegetables has involved a considerable modification in our farm arrangements, which must now aim at the production of that class of vegetables best suited for vegetarian dishes. This will take some time, however, as we have only about fifteen acres arable land."

"Then as to the improvements of service?"

"Well, this has been pretty general everywhere. The old institutional crockery is being replaced by the ordinary table ware of the average English cottage home. Then, again, there has been a cutting up of the large long tables, admitting of some classification not only on a diet basis, but also on a personal one. It has been found by this means that more individual attention is likely to be given than when large numbers of people are sitting in long rows. The general aim in the treatment of the patients as a whole is to reduce as far as possible the chasm separating institutional life from that of their own homes. To this end uniformity of clothing has been abandoned, so that when patients walk out, as they do only in small numbers at a time, they are not stared at by the public."

### The Mess Room Service Improved.

"The improved service is shared in by the staff?"

"Yes. The idea is to increase the attractions of mental work for attendants and nurses, and to get the best type of men and women for the work. Formerly each member of the staff took possession of his weekly allowance of butter and cheese, etc. For some reason this was supposed to prevent grumbling, but it made the weight of food, rather than the meal, the unit of value. There was a large table and one carver, with the result that the last man served got little and got it cold. There was no chance of sociability. It simply meant a scramble to get sufficient food down in the allotted time. The small-table system does away with all this. Each table has sitting capacity of five or six, and is presided over by the senior attendant or nurse, who carves for the same people each day. When this change was proposed it was objected that the small tables with separate joints of meat would mean wasteful carving, because so many more would have to carve who were inexpert; this, however, is not found to be the case."

"The staff has appreciated the change?"

"Yes, greatly. You see there are two messes, for each of which half an hour is allowed. In each ward there is a charge attendant and his or her deputy. The charge carves for the first mess and the deputy for the second mess. The same ward attendants dine at the same tables, and, as I have said, individual tastes can be gratified. Finally, although the set diets are in the experimental stage only, we have established our case for improving the quality, variety, and service of food without increasing the cost, and an extended development may no doubt be expected."

